# Does photobiomodulation influence ageing?

**DOI:** 10.18632/aging.101556

**Published:** 2018-09-15

**Authors:** John Mitrofanis, Glen Jeffery

**Affiliations:** 1 Department of Anatomy F13, University of Sydney, Sydney 2006, Australia; 2 Institute of Ophthalmology, University College London, London, England

**Keywords:** gliosis, red and infrared light, central nervous system

Mitochondria play key roles in regulating the ageing process. When their membrane potential and function declines, their production of adenosine triphosphate (ATP) reduces and they can signal cell death. This is particularly marked in the energy demanding central nervous system, where the neurons and glia, undergo some key structural and functional changes during ageing. For the neurons, there is a progressive decline in their numbers, due to an intrinsic accumulation of aberrant proteins and free radicals, genetic mutations and/or mitochondrial defects [1,2]. The glial cells, by contrast, undergo a striking increase in their number and activity, reflective of an inflammatory response due to the loss of neurons (Lynch et al. 2010). Over the years, many have sought ways to alter the course of these cellular changes, to slow the ageing process and improve quality of later. From genetic manipulations to changes in diet and from using pharmaceuticals and antioxidants to incorporating more exercise and lowering cardiovascular risk factors, all have attempted to extend life and reduce the adverse impact of age [1,2].

Recently, photobiomodulation, the application of red to infrared light (λ=600-1000nm) on body tissues has been reported to alter the course of aged decline. These wavelengths are absorbed by cytochrome c oxidase, the rate limiting enzyme in mitochondrial respiration, increasing its activity along with mitochondrial membrane potential and ATP production [3,4] (Karu 2010; Gkotsi et al. 2014). For the neurons, photobiomodulation improves function, as measured by electroretinograms, in the retina of aged mice (Sivapathasuntharam et al. 2017), together with reducing cell death in a range of experimental pathologies in the brain [4–7]. For the glial cells, photobiomodulation reduces their hyperactivity, by decreasing their proliferation and levels of key structural proteins associated with decline (eg, vimentin, GFAP), oxidative stress (eg, acrolein) and inflammation (eg, α-tumour necrosis factor, complement component C3) in the retina [3] (Begum et al. 2013; Gkotsi et al. 2014; Sivapathasuntharam et al. 2017) and brain (El Massri et al. 2018) of aged mice.

The precise mechanisms used by photobiomodulation are unclear. Mitochondrial and physiological functions are improved, but increased ATP production alone is unlikely to underpin the physiological improvement, as this is relatively temporary. Hence, there are likely to be cascades of signalling between mitochondria and other structures including the nucleus and endoplasmic reticulum that have a wide ranging impact on metabolism that sustain longer term positive changes. For the neurons, several studies have reported that photobiomodulation activates various transcription factors leading to the expression of stimulatory and protective genes related to beneficial cellular features, for example neurogenesis, synaptogenesis and an increase in neurotrophic growth factors [4]. For the glial cells, the mechanisms are less clear. Photobiomodulation-induced reductions in hyperactivity may be secondary to the beneficial effects seen in neurons, but there could be a primary effect also, that photobiomodulation stimulates glial cells directly to reduce the inflammatory response (El Massri et al. 2018).

A key issue for consideration at this point is whether the photobiomodulation-induced benefits seen in the animal models of ageing can be translated to humans. One problem would be method of application, given the large size of the human brain. Photobiomodulation has been reported to penetrate 20-30mm through a range of body tissues, from bone to brain [4–6]. Hence, from a transcranial approach, photobiomodulation would only reach cortical layers of the brain (<10mm), but it would penetrate the retina. For deeper structures of the brain (>30mm), such as the brainstem and hypothalamus, photobiomodulation may mediate benefits indirectly via the circulation, perhaps via cytokine signalling [5,6]. There is evidence that changes in mitochondrial function in one part of the body can influence those at other locations, and improvements in retinal pathology have been reported after photobiomodulation treatment away from the eyes [8]. However, data here are very limited.

In conclusion, photobiomodulation has been shown to alter the course of ageing in the central nervous system, by improving the survival and function of neurons and reducing gliosis and inflammation. These results in the laboratory are ripe for translation to the clinic, to determine whether this treatment effectively slows ageing in humans. Some of the key advantages of photobiomodulation therapy relate to its economy and safety, as it can be delivered with commercially available light emitting devices at energies well within the human safety range. Moreover, a major strength of this therapy is that it can offer a potential clinical application where there is little alternative available.
